# Validity and reliability of the hearing handicap inventory for adults

**DOI:** 10.1590/S1808-86942011000400005

**Published:** 2015-10-19

**Authors:** Camila Piccini Aiello, Ivanildo Inácio de Lima, Deborah Viviane Ferrari

**Affiliations:** 1 Speech and hearing therapist. MSc student in the Graduate Program of the Speech and Hearing Department of the School of Dentistry - University of São Paulo, FOB-USP, Bauru, SP, Brazil; 2 Speech and hearing therapist. MSc student in the Graduate Program of the Speech and Hearing Department of the School of Dentistry - University of São Paulo, FOB-USP, Bauru, SP, Brazil; 3 PhD in Neurosciences and Behavior - Institute of Psychology of the University of São Paulo, Professor at the Department of Speech and Hearing Therapy of the School of Dentistry - University of São Paulo, FOB-USP, Bauru, SP, Brazil

**Keywords:** adult, hearing loss, questionnaires, reproducibility of results

## Abstract

**Abstract:**

The Hearing Handicap Inventory for Adults (HHIA) translated into Brazilian Portuguese has been used for clinical practice and research purposes; however, information regarding its ease of reading and psychometric properties are still lacking.

**Aim:**

To evaluate the ease of reading and psychometric properties of the Brazilian translation of this tool, including its validity and reliability.

**Materials and Methods:**

Prospective study. The questionnaire was applied to 30 normal hearing (Group A) and 113 hearing impaired (Group B) persons. Thirty two participants (group B) answered the questionnaire a second time. The Flesch readability index was calculated for each item in the questionnaire. The internal consistency, test-retest reliability and discriminant validity were evaluated.

**Results:**

Flesch's scores showed that the questionnaire was easy to read. Cronbach's alpha and Pearson's correlation showed high internal consistency. There was no significant difference between test and retest scores. Besides, correlation between these two scores was also high and significant. Student t test indicated significant difference between scores for groups A and B (discriminant validity).

**Conclusion:**

The Hearing Handicap Inventory for Adults translated into Brazilian Portuguese maintained the reliability and validity of the English version. Further studies are needed to determine the convergent validity and construct validity for this instrument.

## INTRODUCTION

Today in health care there is a growing need to systematically and objectively measure, show and document the benefits or results from an intervention - the so-called “result assessment”.

The result assessment can be used to provide data to government entities concerning the use of financial resources, to show certification agents the effectiveness of audiologic services rendered, to show patients and family members the changes which happened because of the intervention, to validate clinical decisions concerning individual hearing aid sound amplification device selection and fitting practices, to determine what is being done correctly and areas which need improvements in the service, as well as to establish good practices for the profession^1^.

One of the points of interest is to assess intervention results in the fields of activity limitation and participation restriction. Activity limitation is characterized as the consequences in functional performance impairment, in other words, in the execution of a given task or action. Participation restriction (handicap) concerns the involvement in life situations and it reflects the individual's adaptation to the environment as a result of the hearing loss and the handicap^2^. In order to do that, it is necessary to determine the difficulties the patient experiences before and after the intervention. In regards to participation restriction, different questionnaires have been developed for this purpose, including the Hearing Handicap Inventory for Adults - HHIA^3^. This questionnaire is based on a modified version of the Hearing Handicap Inventory for the Elderly - HHIE, to be used with individuals aged below 65 years^4^. For such, three questions from the original HHIE questionnaire have been modified in order to include the items created to identify the effects of hearing loss on occupational issues, since younger adults live through this situation more often than the elderly, who are usually already retired^3^. The original HHIA version, in English, has high internal consistency of its questions, test-retest reliability and low standard error^3,^^5,^^6^.

The HHIA questionnaire has been going through adaptations and validations for other languages. The Italian version of the HHIA kept the validity and reliability of the original version, being considered of high relevance in order to establish the non-auditory symptoms of the hearing impaired from that country^7^.

The HHIA questionnaire was translated into Brazilian Portuguese^8^ and has been used both in the audiology clinic as well as in research in order to quantify the handicap of a population, to assess the benefits of using the ISAD or that of intervention programs for people with noise-induced hearing loss^8^, ^9^, ^10^, ^11^, ^12^. Nonetheless, data on the validity and reliability of the HHIA translated into Brazilian Portuguese has not been reported in the literature, and this is a limiting factor in regards of the usefulness of the instrument for use in research or to document clinical interventions.

The goal of the present study was to assess the psychometric properties of the Adult Auditory Handicap questionnaire translated into the Brazilian Portuguese, including its validity and reliability, as well as its ease of reading and application in adult normal hearing and hearing impaired individuals.

## MATERIALS AND METHODS

Prospective study held in the Audiology Clinic of the Dentistry School of Baurú - University of São Paulo (FOB/USP), approved by the Ethics in Research Committee of this Institution (process # 05/2009).

All 143 participants volunteered and signed the Informed Consent Form. The participants were broken down into the two groups described below.

### Group A

Made up of 30 normal hearing adults (15 women and 15 men), with ages between 20 and 60 years (mean age of 38.5 years). None of the participants had a past or complained of hearing disorders, and they also did not have any external ear or tympanic membrane disorder found upon ear inspection. All the individuals were submitted to audiometric screening (carried out in a sound-treated booth, and the 250 to 8000 Hz frequencies were studied, with a presentation level equal to 25 dB HL). All the individuals could read and they did not have previous knowledge about the Auditory Handicap Inventory for Adults.

### Group B^1^

Data from this group can be found on Table 1. Made up of 113 individuals (52 women and 61 men) with ages varying between 21 and 64 years (mean age of 53.6 years), with bilateral post-lingual sensorineural hearing loss, symmetrical (n=85) or asymmetrical (n=28). The ISO mean (frequency of 500, 1k, 2k and 4k Hz) of the audiometric thresholds from the best ear varied from 26.25 to 92.5 dB HL (mean of 49.7 dB HL). According to the ISO mean value from the best ear, the hearing losses were classified as mild (n=32; 28.3%), moderate (n=62; 54.8%); severe (n=13; 11.5%) and profound (n=6; 5.3%).

**Table 1:** Data on the participants' hearing loss

		# of individuals	Total
	26 to 40	32	
Threshold mean dB HL (500, 1k, 2k, 4kHz)			
	41 to 60	62	113
	61 to 80	13	
	> 80	6	
	15 to 30	14	
Speech recognition threshold - SRT (dB HL)			
	35 to 50	62	107
	55 to 70	24	
	75 to 90	7	
	Up to 12 m.	2	
Hearing impairment duration (months)	12 to 36 m.	11	
	37 to 60 m.	11	106
	61 to 120 m.	26	
	> 120 m.	56	

It must be stressed that seven participants were not able to report on the duration of the hearing loss. Six participants were not able to undergo the speech recognition threshold test because of their hearing loss severity (Table 1).

Information about educational level was obtained for 108 participants, and they had incomplete (n=47; 43.5%) and complete (n=10; 9.2%) basic education; incomplete (n=10; 9.2%) and complete (n=30; 27.7%) high school education; and complete higher education (n=8; 7.4%). Three individuals (2.7%) were illiterate.

Socioeconomical information was obtained for 112 participants based on the Socioeconomical Instrumental Classification protocol present in the patients' charts^13^. The following classifications were checked: low inferior (n=13; 11,6%), low superior (n=76; 67.8%), medium inferior (n=19; 16.9%) and medium (n=4; 3.5%).

All Group B participants were regularly enrolled in the FOB-USP audiology clinic and did not have previous experience with the ISAD.

We used the Adult Auditory Handicap questionnaire translated into Brazilian Portuguese^8^ (Attachment 1). This questionnaire is made up of 25 questions broken down into two subscales: social (12 questions, which measure the effects of hearing loss in different social situations) and emotional (13 questions, which estimate the behavior and emotional responses of the individual in relation to his hearing loss). All questions are identified according to the scale to which they belong. For each question there are three possible answers: “yes” (equal to 4 points), “sometimes” (worth 2 points) and “no” (equal to 0 points). The scoring was carried out manually, when we calculated the total score (summation of the points for the 25 questions), as well as to the emotional and social subscales, separately. The total HHIA score can vary between 0 and 100; the social scale score can vary between 0 and 48 and the emotional scale can vary between 0 and 52. Higher values indicate a greater perception of the auditory handicap.

Initially, we calculated the Flesch Reading Ease Index (FREI) for each question in the questionnaire in order to assess the level of text reading difficulty based on the length of the words and phrases^14^. FREI assesses the degree of ease of reading the texts in a percentage scale. The formula includes sentence size and the number of syllables in a sample of 100 words. The higher the value, the greater the ease of reading the text assessed and the lower the educational level necessary for its understanding. The scale is made up of seven levels, varying from “very easy” (score between 90 and 100%) to “very difficult “ (scores between 0 and 30%)^15^.

For Group A, the questionnaire was applied in the form of pen and paper, and the individual was asked to read the 25 questions and check the answer he/she thought more appropriate. The time it took the participant to fill out the questionnaire was calculated. At the end of it, the examiner interviewed the participant in order to check his/her perceptions on the ease of understanding and filling out the questionnaire.

For Group B, the HHIA questionnaire was applied in the form of an interview after audiologic diagnosis. The examiner read the 25 questions together with the participant who was asked to check the answer he/she thought more adequate. For 32 individuals (13 women and 19 men) from Group B, the Hearing Handicap Inventory for Adults was applied a second time, again in the form of an interview, at least two weeks after the first application, but prior to the ISAD fitting, and this time interval was considered acceptable to minimize the issue of memory for the questionnaire items which could contribute to a strong correlation between the two applications.

The statistical analysis was carried out by means of the Stata software. In regards of reliability, for Group B participants, the internal consistence of the questionnaire was measured by the Cronbach's alpha, which was also calculated when each item was removed from the questionnaire. The Pearson's correlation coefficient was utilized in order to study the relationship between the total score and the social and emotional subscales, as well as the correlation between the two subscales.

In order to assess the questionnaire's test and retest variability, we calculated the Pearson's correlation coefficient between the total score and that of the social and emotional subscales between the first and second application of the questionnaire. The existence of a significant difference between the scores obtained in the two applications was analyzed by means of the paired t-test. We also calculated the standard error and the 95% confidence interval.

In order to check the discriminant validity, the questionnaire scores obtained for Group A and for 32 participants from Group B were compared by means of the Student t test. In all the analyses we adopted a 5% significance level.

## RESULTS

The application of the Flesch Reading Ease Index showed 13 questions which were considered very easy or reasonably easy, six questions were considered standard, five were considered reasonably difficult (E-2, E-5, S-15, E-17, S-21) and one question (S-10) was considered very difficult.

The scores obtained from the questionnaire for Group A participants was 1,1. For this group, the time taken to fill out the questionnaire varied between 1.30 and 4.13 minutes (mean of 2.28 minutes). When questioned about the level of difficulty found in the filling out of the questionnaire, 29 individuals (96%) reported that it was easy to understand.

**Figure 1:**
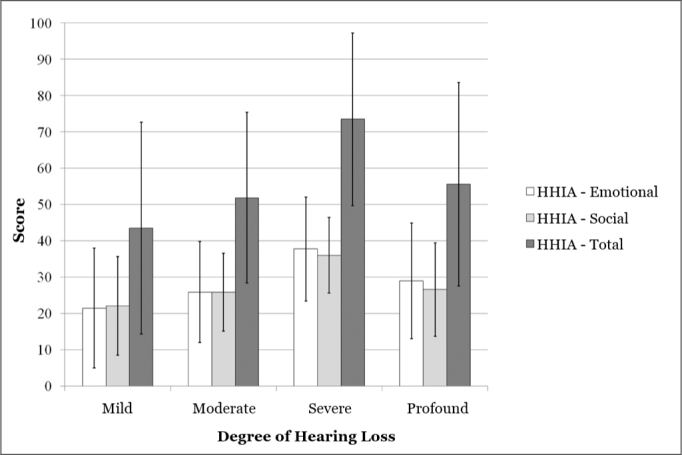
Median and standard deviation of the total score and that from the social and emotional subscales for Group B, in accordance with the hearing loss in the best ear (n=113).

For Group B participants, the mean scores and standard deviation values obtained from the HHIA questionnaire was equal to 52.2 ± 26.6 (total); 25.9 ± 12.1 (social) and 26.3 ± 15.3 (emotional). Figure 1 shows the mean and standard deviation for the HHIA score for Group B participants, according to the degree of hearing loss (Fig. 1).

HHIA's internal consistency using the Cronbach's alpha was 0.92 (total score), 0.91 (social) and 0.84 (emotional). When each one of the items was taken off the scale, the Cronbach's alpha varied between 0.93 and 0.94. the Pearson's correlation coefficient and the significance level between the total score and that of the social and emotional subscales for Group B participants can be found on Table 2.

**Table 2:** Pearson's correlation coefficient between the total score and that from the social and emotional subscales of the HHIA for Group B (n=113).

	HHIA - Social	HHIA - Total
	r = 0,86	r = 0,97
HHIA - Emotional	*p* = 0,00	*p* = 0,00
		r = 0,96
HHIA - Social	-	*p* = 0,00

The questionnaire's test and retest variability results, calculated from a sample of 32 participants from Group B can be found on Table 3.

**Table 3:** Mean and standard deviation values from the scores obtained; how significant is the comparison between the means and correlation of the results from the test and retest for Group B participants (n=32).

	Test	Retest	Paired t Test *p* value	Pearson's correlation (r)
Total HHIA	44,8 ± 27,9	45,9 ± 27	0,72	0,81U+0002A
Social HHIA	22,4 ± 12,4	22,9± 12,7	0,73	0,79U+0002A
	22,4 ± 16,3	23 ± 14,7	0,76	0,78U+0002A

* *p*=0,00

The discriminant validity was checked by the comparison between results from Groups A and B, as per depicted on Table 4.

**Table 4:** Comparison between the mean values and the deviations of the scores obtained for Group A (n=30) and for Group B (n=113).

	Group A	Group B	t Student test *p* value
Total HHIA	1,1 ± 2,3	52,2 ± 26,6	0,00
Social HHIA	0,4 ± 1,1	25,9± 12,1	0,00
Emotional HHIA	0,6 ± 1,4	26,2 ± 15,3	0,00

## DISCUSSION

In general, the results from the reading ease index of the questions which make up the HHIA questionnaire in Portuguese are of ease reading, nonetheless, it has been suggested that question S-10 be remade. One Group A participant considered the language utilized in the instrument as being too technical. The time it took to fill out the questionnaire, in the form of pen and paper, indicated that it can be used in clinical practice, without requiring much clinical time. It must be stressed that most Group A participants were individuals with high education, which not always represents the true population seeking the public health-care system.

For Group A, we obtained the lowest scores in the HHIA questionnaire (Table 4). The social subscale score varied between 0 and 4 points (mean = 0.47) and the emotional subscale varied between 0 and 6 points (mean = 0.67). The total score varied between 0 and 10 points (mean = 1.13). Such results indicated that no handicap was perceived, which was expected since the participants were normal hearing people.

Reliability is defined as the degree in which the measured result reflects the true result. In the present study, reliability was measured by the internal consistency and the test-retest consistency.

The internal consistency checks the degree in which a group of observed variables is measuring a given construct. The minimum acceptable value for the Chronbach' alpha coefficient for internal consistency is equal to 0.7^0^^3^. The general reliability of the HHIA translated into Brazilian Portuguese measured by the Cronbach's alpha was 0.92. Even when each one of the items is removed from the scale, Cronbach's alpha remained high, varying between 0.93 and 0.94. These results are similar to those from the original version - in English, and also from the Italian version of the instrument^5,^^7^.

We found strong and significant correlations between the total score and that from the social and emotional subscales, as well as between the subscales (Table 2), indicating that in the translation of the HHIA into Portuguese there are associations between the constructs which were measured in each subscale with the total score of the questionnaire. In the original version in English, we observed correlations between 0.84 and 0.9^6^,^5^.

In regards of the test-retest consistency, it is possible to notice that the differences between the mean values obtained between the first and second times the questionnaire was applied varied between 0.5 (social subscale) and 1.1 (total score) and these were not significant (Table 3). We also noticed that the standard deviations found in the test and retest were very similar, indicating that the variation in score between the participants was similar from one time the questionnaire was employed to the other. And finally, strong and statistically significant correlations were found between the first and the second time the questionnaire was employed. This data is in agreement with what was found for the original version, in English and for validation in Italian, when we found correlations around 0.[Bibr bib9][Bibr bib3][Bibr bib7]. The test-retest reliability shows the stability of an instrument in the long run. This means that the HHIA translated into Brazilian Portuguese may produce valid and consistent results from one time it is used to another. One of the HHIA' goals is to serve as criterion to document the effects of treatment, including the benefits of using an ISAD^16,^^17^, throughout time, this result is extremely important.

In regards of the discriminant validity, the hypothesis adopted was the one that the questionnaire shows discriminant validity, the scores from individuals with hearing loss would differ from the scores from normal hearing people. As we can see on Table 4, Group B individuals had significantly higher scores in the HHIA (indicating a higher handicap) when compared to those from Group A. This happened both from the total scores and from the subscales such as those from comparing each question from the questionnaire individually. However, it must be stressed that in the present study it was not possible to match the socio-demographic data from Groups A and B participants, and this may have influenced the results.

## CONCLUSION

The results from this study show that the Brazilian Portuguese version of the Hearing Handicap Inventory for Adults maintains the validity and reliability of its original version. Other studies are needed in order to establish the converging validity and that of the construct of this instrument.

**Table:** 

**Attachment 1**: THE HEARING HANDICAP INVENTORY FOR ADULTS (HHIA)				
(Newman, Weinstein, Jacobson e Hug 1990)				
(Adaptation for Brazilian Portuguese - Almeida, 1998)				
Name: _________________________________________________ Date: __________________				
Instructions: The following questionnaire has 25 questions. You must choose only one answer for each question, checking (X) the one you find more adequate. Some questions are similar, but in reality they have subtle differences which enable a better assessment of the answers. There is no right or wrong answer. You should check the one you find most adequate to your case or situation.				
		Yes (4)	Sometimes (2)	No (0)
S-1	Does your hearing difficulty make you use the phone less often than you'd like?			
E-2	Does your hearing difficulty make you feel embarrassed or out of place when you are introduced to strangers?			
S-3	Does your hearing difficulty make you avoid groups of people?			
E-4	Does your hearing difficulty make you touchy?			
E-5	Does your hearing difficulty make you feel frustrated or unhappy when talking to people of your family?			
S-6	Does your hearing impairment cause any other difficulties when you go to a party or social meeting?			
E-7	Does your hearing difficulty make you frustrated when talking to work mates?			
S-8	Do you feel hearing difficulties when you go to the movies or the theater?			
E-9	Do you feel harmed or down because of your hearing difficulty?			
S-10	Does you hearing impairment cause difficulties when you visit friends, relatives or neighbors?			
S-11	Does your hearing difficulty cause you problems to hear/understand work mates?			
E-12	Does your hearing difficulty make you nervous?			
S-13	Does your hearing difficulty make you visit friends, relatives or neighbors less often than you'd like to?			
E-14	Does your hearing difficulty make you argue or fight with your family?			
S-15	Does your hearing difficulty cause you trouble to watch TV or listen to the radio?			
S-16	Does your hearing difficulty make you go out shopping less often than you'd like to?			
E-17	Does your hearing difficulty make you annoyed or unhappy?			
E-18	Does your hearing difficulty make you prefer to be alone?			
S-19	Does your hearing difficulty make you want to talk less to the people in your family?			
E-20	Do you think that your hearing difficulty reduces or limits your personal or social life somehow?			
S-21	Does your hearing difficulty cause you trouble when you are in a restaurant with family or friends?			
E-22	Does your hearing difficulty make you feel sad or depressed?			
S-23	Does your hearing difficulty make you watch less TV or listen to the radio less often than you'd like to?			
E-24	Does your hearing difficulty make you feel embarrassed or less comfortable when you talk to friends?			
E-25	Does your hearing difficulty make you feel isolated or left aside within a group of people?			
FOR PHYSICIAN USE: Total score: __________ Subtotal E: ________ S: ________				

## Footnotes

1 This group participated in a previous study from the authors called “Audiometric correlations of the HHIA questionnaire”. CEFAC journal, prelo.
